# Family support for children with learning disabilities to attain good academic performance: A qualitative study

**DOI:** 10.51866/oa.529

**Published:** 2024-04-24

**Authors:** Uraiwan Tiengsomboon, Varisara Luvira

**Affiliations:** 1 MD, Dip. Thai Board of Family Medicine, Department of Community, Family, and Occupational Medicine, Faculty of Medicine, Khon Kaen University, Khon Kaen, Thailand. Email: varisara@kku.ac.th; 2 BNS, MNS, PhD Candidate in Community Health Development, Department of Community, Family, and Occupational Medicine, Faculty of Medicine, Khon Kaen University, Khon Kaen, Thailand.

**Keywords:** Learning disability, Parenting, Educational achievement

## Abstract

**Introduction:**

Learning disabilities can cause poor academic performance in children, which may impact their futures. This study aimed to investigate how primary caregivers care for school-aged children with learning disabilities but with good academic achievement.

**Methods:**

In this qualitative study, in-depth interviews were conducted among primary caregivers of school-aged children with learning disabilities who were attending schools in Sisaket Province, were aged 6–12 years and achieved good academic performance. Twenty-one caregivers were interviewed regarding the care of their children. The contents of the interviews were analysed.

**Results:**

Two major themes concerning the provision of familial support for children with learning disabilities to achieve good academic performance emerged: (1) understanding and modifying the care provided to children with learning disabilities and (2) facilitating and promoting children’s learning.

**Conclusion:**

Families and caregivers of children with learning disabilities must have a comprehensive understanding of the disorder to assist with skill development and provide emotional support.

## Introduction

Learning disabilities are a group of heterogeneous disorders that can impact the acquisition, organisation, retention, comprehension or application of verbal or nonverbal information.^1^ Reading, writing and mathematical skills can all be affected by learning disabilities. According to the Diagnostic and Statistical Manual of Mental Disorders, Fifth Edition, the estimated global prevalence of all learning disorders ranges from 5% to 15%.^2^ These disorders interfere significantly with academic performance or daily living activities while maintaining an average or above-average intelligence quotient.^3^ Children with learning disabilities are more likely to repeat grades or leave school than average students.^4^ The effects of learning disabilities may persist into adulthood, as reading, writing and mathematical skills are typically necessary for performing routine daily activities. Those affected may also encounter limited employment opportunities. 1 Remedial intervention using behavioural and medical techniques to enhance the functioning of individuals with learning disabilities is the cornerstone of treatment. The treatment of children with learning disabilities depends on the continuous coordination and collaboration of multidisciplinary teams.^5^ Educators who specialise in working with children with special needs or talents are typically responsible for administering remediation.^1^ The key principles of effective intervention for educators to apply to children with learning disabilities are based on evidence-based intervention programmes characterised by explicitness, comprehensiveness, individualisation and intensity. Furthermore, early intervention has greater efficacy in promoting development. For instance, the efficacy of an intervention is greater when implemented in grade 1 or 2 than in grade ^3,6^

Children with learning disabilities may exhibit behaviours that are similar to those of their typically developing peers. Parents usually exhibit unfavourable attitudes and responses towards their children's diagnosis of learning disability, including rejection, denial, overprotection and hopelessness.^7^ Inadequate knowledge, adaptation difficulties and parental burden result from these attitudes and responses.^7^ Therefore, parents refute their children’s learning difficulties when instructors or others attempt to identify them.^8^ As the primary caregivers of their children, parents play a crucial role in enhancing and facilitating their children’s academic achievements. A nurturing and supportive home environment can effectively and positively influence the learning behaviour of children with learning disabilities.^7,9^ In addition, parents play a substantial role in engaging in discussions and consultations with teachers at school to collaboratively explore strategies for comprehending and nurturing their children’s abilities within an appropriate educational environment.^9^

Children with learning disabilities have learning difficulties but can develop their learning potential to be able to function effectively in society. Academic achievement is therefore an essential indicator of the effectiveness of learning and development. At present, Thai children with learning disabilities can pursue inclusive education. Educational institutions in the country implement an individualised education programme (IEP) as part of their academic framework. The IEP serves as the primary tool within the educational system for the development, guidance and implementation of transition plans that aim to facilitate successful outcomes in postsecondary settings.^10^ Children with learning disabilities often depend on specialised instruction to develop and enhance skills that are affected by their condition, in which active participation of their family is crucial. Family caregivers possess an advantageous position to facilitate their children’s learning through the provision of individualised attention and prompt implementation of necessary adjustments. To improve their children’s academic performance, family caregivers are encouraged to frequently participate in tutoring their children’s learning activities and implementing interventions at home. In addition, parents should have a thorough understanding and effective management of the emotional, behavioural and learning demands of children with learning disabilities.^11^

In this study, the researchers studied Sisaket Province, which is located in Thailand’s northeastern region. In Sisaket Province, rural life persists. A mixed ethnic and religious makeup reflects the province's uniqueness. Social structure of the province is very coherent due to these traits. Familial and close relationships influence family dynamics. In the province, only 1.26% of children with learning disabilities have shown good academic performance with a minimum overall grade point average of 3.00. The researchers were thus interested in examining the attributes of family care that lead to good academic performance in children with learning disabilities. They focused on studying school-aged children (aged 6–12 years). Learning difficulties can significantly influence children's academic achievement. Children at this age may struggle in several areas of development, which might impact on their learning abilities as they transition into adolescence and adulthood. The focus of the study was to understand the nature of the care given to these children. This study aimed to investigate how primary caregivers care for school-aged children with learning disabilities but with good academic achievement. This issue has not been explored in previous studies. The findings are anticipated to serve as a guideline for rearing children with learning disabilities so that they can have a solid foundation and the necessary skills to function in society.

## Methods

This qualitative study examined the attributes of family care that contribute to good academic performance in children with learning disabilities. It investigated how primary caregivers care for school-aged children with learning disabilities but with good academic achievement. The study included primary caregivers of children aged 6–12 years who had been diagnosed with learning disabilities. The caregivers included parents or grandparents depending on the context of each family concerning who had the primary responsibility for caring for such children. The children were attending schools in Sisaket Province and had demonstrated good academic performance based on a minimum overall grade point average of 3.00 and the fulfilment of the criteria outlined in the IEP. Purposive sampling was employed as the recruitment method by submitting a formal request to the Office of Educational Administration seeking permission to obtain information regarding the academic performance of children with learning disabilities. Once the chosen group of students was identified, the researchers coordinated with the school and class teacher to begin contact with parents who were interested in participating in the study. The study collected data at the children’s schools from February to May 2023. The number of primary caregivers to be interviewed was determined based on information saturation, meaning that no additional useful information was obtained from further interviews.^12^ This point was reached after interviewing 21 primary caregivers from 11 schools, where children with learning disabilities study alongside their peers in general classrooms. The researchers conducted in-depth interviews with the primary caregivers at the educational institutions attended by their children. Each interview lasted 30–45 min. The number of interviews varied based on the saturation of data, typically ranging from one to two interviews. The researchers were not directly engaged in the academic investigations of the children at their schools.

During the interviews, an in-depth interview guide, which encompassed inquiries regarding demographic information and the characteristics of family care, was utilised. The family care section covered key questions related to raising children, promoting their learning and development, understanding their learning abilities and setting expectations for their academic achievement. Informed consent for participation was then obtained. Researchers directly gathered all data through written documentation and voice recordings. The research team comprised clinicians skilled in both teaching and conducting qualitative research. The interviewers had no prior relationship with the participants. Two transcribers transcribed the interviews verbatim, ensuring that all identifiers were anonymised. During the interviews, a note-taker recorded nonverbal indicators and identified the participants for subsequent transcription. Participant conduct during interviews was recorded via field recordings.

Content analysis was performed. Data were initially analysed by extracting the indepth interview records verbatim. Details surrounding the interviews were also documented. The extracts were additionally validated via triangulation to enhance the credibility and validity of the findings.^13^ In this study, triangulation entailed gathering data on multiple occasions at various time points to examine their consistency across different collection instances. Subsequently, the researchers proceeded to analyse the data obtained from the interviews through a systematic process of organising, classifying and categorising them within the established research framework. The researchers carefully chose the words that were the most meaningful and effective words to convey the content. Two researchers independently examined themes and codes. All conflicts were successfully resolved through collaborative discussions aimed at reaching a mutually agreed-upon conclusion.

## Results

This study included a total of 21 primary caregivers of children with learning disabilities. There were 18 women and three men aged from 29 to 64 years (Table 1).

**Table 1:** Participant profiles.

Participant	Age of in-care children, year	Sex of in-care children	Marital status	Family structure	Educational level	Relationship with children
P1	10	Male	Married	Extended family	Junior high school	Paternal grandmother
P2	10	Female	Married	Nuclear family	Senior high school	Aunt
P3	9	Male	Married	Nuclear family	Bachelor’s degree	Mother
P4	10	Male	Married	Extended family	Diploma	Father
P5	9	Male	Married	Extended family	Grade 4	Maternal grandmother
P6	9	Female	Married	Extended family	Diploma	Paternal grandmother
P7	9	Male	Married	Nuclear family	Bachelor’s degree	Mother
P8	12	Female	Married	Nuclear family	Bachelor’s degree	Mother
P9	8	Female	Married	Extended family	Bachelor’s degree	Mother
P10	10	Male	Married	Extended family	Bachelor’s degree	Mother
P11	12	Male	Married	Extended family	Diploma	Paternal grandfather
P12	9	Male	Divorced	Extended family	Bachelors degree	Mother
P13	11	Male	Married	Extended family	Bachelors degree	Mother
P14	11	Male	Divorce	Extended family	Bachelors degree	Mother
P15	12	Female	Married	Extended family	Fourth grade	Maternal grandmother
P16	12	Male	Widowed	Extended family	Fourth grade 4	Maternal grandmother
P17	11	Male	Married	Extended family	Bachelor’s degree	Mother
P18	9	Female	Separated	Extended family	High vocational certificate	Mother
P19	11	Male	Married	Extended family	Bachelor’s degree	Mother
P20	11	Male	Married	Nuclear family	Bachelor’s degree	Mother
P21	12	Male	Married	Extended family	Diploma	Paternal grandmother

The analysis revealed the emergence of two main themes about the provision of familial support for children with learning disabilities to achieve good academic performance. **Table 2** summarises the two themes and nine subthemes.

**Table 2:** Themes and subthemes.

Theme	Subtheme	Representative quote
1. Understanding and modifying the care provided to children with learning disabilities	1.1 Embracing a mindset that fosters acceptance and comprehension of children’s learning disabilities	“At first, I thought my child had some kind of problem that made him hate school. But now I know that he has a learning problem. So, I open my heart to accept him as he is. I’m ready for his teachers and the school to help him learn as much as possible. I never felt ashamed because I loved him so much. Our family accepts that he has a learning disability and is dedicated to giving him the best possible development. About a year or two ago, he didn’t understand anything. He couldn’t read or write, and he couldn’t add or subtract. But he’s getting better at it now, which makes me happy and encouraged”. (P7)
	1.2 Establishing children’s future goals and managing expectations for their development	“I really want her to get as high an education as possible. I want her to be able to continue her schooling like everyone else. I want her to finish school and get a job just like everyone else. I also want her to be able to take care of herself as she grows up. But I don’t think I can hope for much. I want her to be happy, so let her pick what she wants to do”. (P8)
	1.3 Developing a structured framework of discipline for children	‘I taught him how to behave and gave him rules to follow. There is a time limit for him to do his homework, help take care of his younger brother and help me finish housework. After that, he can play with his cell phone for up to 2 h, and then he must go to bed by 9 p.m. at the latest. I taught him to be organised, like picking up his toys after playing and putting things in order. I also taught him to wash and iron his own clothes so he could be able to help himself’. (P10)
	1.4 Restraining oneself and managing one’s feelings	“I try not to have negative emotional reactions to him. When I teach him, I have to try to stay very calm and not let myself get angry when he doesn’t get it. I teach him with an understanding of him, praise him and encourage him”. (P19)
2. Facilitating and promoting childrens learning	2.1 Providing special affection and attention	“I teach him to read more books every day, two pages a day, and three to four pages on Saturdays and Sundays, especially if I have time for him and can pay a lot of attention to him on a daily basis. I will see how he progresses. From the start, he was unable to read and wrote inverted. I’ve seen everything. But now my son is more interested in learning and can read better, even though he still writes some letters wrongly. But I told him, ‘I believe in you’, ‘You can do it’ and “Try your best, and I’ll always be there to cheer you on”. Rewarding and praising him will encourage him to be more attentive and responsible in his studies”. (P13) ‘My wife and I work together to help our child with reading, writing and math. Practise every day, keep doing it every day and be persistent. We buy books to give our child a full set of learning tools to help her practise learning and let our child do it herself, while we watch and encourage her’. (P8)
	2.2 Fostering a sense of pride in children	“I always tutor him on his homework and teach him to be responsible and complete his assignments. I occasionally let him practise reading, writing or doing assignments on his own. I always tell him, “If you can’t do it, you can always ask me”. He appreciated it when I encouraged him, “You’re good, you can do it”. He smiled whenever he heard that and was more determined”. (P12) ‘I support my son in everything he does, whether it is academics, sports or anything else he likes. I want him to be proud of himself when he does something well’. (P13)
	2.3 Collaborating with educators	“I always ask my son’s teacher to help him. I always use LINE to talk to my child’s teacher about his learning progress and behaviour”. (P4)
	2.4 Instructing with examples	“I initially produced an example for him to view. I’d start by reading, writing or adding numbers for him to see and then let him follow along. He would remember to do more”. (P7)
	2.5 Other family members contributing to the improvement of academic skills	‘Many people in my family helped him with his homework. When I teach by myself, I sometimes get too tired to keep going. Some days, after work, his mother uses video talks to teach him’. (P1) ‘Her bigger brother assists and teaches her homework. He studies well and helps them take care of each other’. (P15)

*Theme 1: Understanding and modifying the care provided to children with learning disabilities*

***Subtheme 1: Embracing a mindset that fosters acceptance and comprehension of children' learning disabilities***

During the interviews, the participants demonstrated a willingness to accept and comprehend that their children had learning disabilities. They possessed an awareness that their children were encountering difficulties and required assistance.

***Subtheme 2: Establishing children’s future goals and managing expectations for their development***

The participants established goals and expectations for their children’s future, which were categorised into academic and life domains. For the academic domain, the participants aspired for their children to achieve their highest potential in their studies. For the life domain, the participants aimed for their children to develop the necessary skills and abilities to thrive and succeed. However, the participants were hesitant to have high expectations.

***Subtheme 3: Developing a structured framework of discipline for children***

The participants established a set of rules encompassing both academic and daily routines, including household chores, with the aim of fostering a sense of responsibility in their children.

***Subtheme 4: Restraining oneself and managing one’s feelings***

Due to the children’s lack of comprehension, teaching children with learning disabilities sometimes caused the participants to experience negative emotions. Therefore, the participants attempted to maintain composure and refrain from expressing such emotions to their children.

*Theme 2: Facilitating and promoting childrens learning*

***Subtheme 1: Providing special affection and attention***

Most participants provided care and promoted their children’s learning by paying special attention to them. This involved training and teaching their children to engage in repetitive tasks, establish regular routines and maintain consistent participation by demonstrating affection and support through physical embraces, verbal expressions of love and compliments. Additionally, the participants provided diverse rewards as incentives.

***Subtheme 2: Fostering a sense of pride in children***

To foster a sense of pride in their children, the participants provided them with opportunities to participate in a variety of activities that aligned with their interests. They encouraged their children to perform these activities step by step until they were able to do so on their own as well as exercise the skills that were lacking while encouraging or praising them for their enhanced abilities.

***Subtheme 3: Collaborating with educators***

The participants communicated with teachers to assist their children’s learning and improve their behaviour.

***Subtheme 4: Instructing with examples***

The participants provided examples of reading, writing and mathematics so that their children could more easily follow along.

***Subtheme 5: Other family members contributing to the improvement of academic skills***

In addition to the participants, who were the primary caretakers of the children, it was discovered that in most families where children with learning disabilities had good academic performance, many family members such as the children’s siblings and parents assisted in promoting the development of various skills in studying even though some families did not live together due to parental migration. Further, video calls were also used to assist children with their homework. This increased the number of family members assisting with childcare, reducing the burden on the primary caregiver.

## Discussion

This study was a qualitative study using indepth interviews with the primary caregivers of children with learning disabilities whose academic performance was good. Parents' attitudes and practices can both contribute to good academic performance for their children who have learning disabilities. When parents understand the condition of their children with learning disabilities, they cultivate an open-minded attitude and acknowledge the challenges associated with their children’s learning difficulties. They also find hope and create goals for their children’s future. They create techniques for their children to improve their learning capacity, while also demonstrating self-restraint and emotional management when caring for and educating them. In practice, parents assist and promote children’s learning by creating a sense of pride in them, collaborating with educators, teaching with examples and encouraging other family members to participate in enhancing children’s academic skills.

This study found that in families where children with learning disabilities achieved good academic performance, the parents demonstrated understanding and modified the care provided to their children with learning disabilities. This finding aligns with a previous report that suggests the importance of providing parental education regarding learning disabilities.^1^ One barrier that limits the progress of children with learning disabilities is the lack of knowledge and negative attitudes displayed by parents. This often leads to rejection, denial, overprotection and a sense of hopelessness.^7^ However, if parents understand accurate information on children with learning difficulties, they can facilitate the optimal development of their children’s learning potential. In this study, the participants emphasised that their aim for the future was for their children to be able to care for themselves when they become adults. Prior research has demonstrated that parents of children with learning disabilities were concerned about their children’s future, which may be uncertain in the professional world and life.^9^ In particular, studying was still essential because it was a prerequisite for integrating successfully into adult society. The group of students had learning disabilities that directly impacted their academic performance. However, the parents of the children in this study still dared to set the highest possible academic objectives for their children. This finding differs from other reports on parents of children with learning disabilities.^7^ Their approach reduced the likelihood that children with learning disabilities would attain advanced academic credentials.^4^ With the courage to have high educational goals and value children’s future lives, the parents and families of the children in this study intended to promote their children’s potential in both academics and daily life. In part, it remained comparable to providing for children without learning disabilities. While the other portion was unique, it was appropriate for children with learning disabilities, the care of whom required greater understanding and effort than that of children in general. In addition, the caregivers in this study were concerned about their children’s future, fearing that they would be unable to engage in society. This finding is consistent with a prior report.^9^ Therefore, daily life skills were also reinforced for the children. Using analogous principles to study skills training both established rules and provided emotional support. However, when teaching was not comprehended by their children, the caregivers sometimes experienced negative emotions. Similarly, a previous study showed that parents could experience emotional strain^5^ in addition to the stress already experienced when caring for children with learning disabilities.^14-16^ Therefore, caregivers must attempt to comprehend their children’s disability. In this study, refraining from expressing negative emotions to their children was beneficial to the academic development of the children.

Herein, the participants were knowledgeable of their children’s learning disabilities. Therefore, they emphasised academic support and improvement. The participants prioritised the importance of teaching repeatedly, encouraging children to regularly practise their study skills, leading by example and maintaining open lines of communication with teachers to collaboratively support their children’s learning. These findings are consistent with previous reports indicating that parents of children with learning disabilities were involved in their children’s education and had a relationship with their children’s schools.^9^ In this study, the caregivers emphasised the significance of providing emotional support for their children. They motivated their children to be more determined to improve their academic skills by encouraging them to take pride in themselves. Recognising that teaching study skills and providing psychological support require more effort for children with learning disabilities than for typically developing children, including learning rules established for these students. This demonstrates the significance and concern given to the education of children, even when they have a learning disability. In addition, having many family members involved facilitates the academic development of children. This is consistent with findings from a previous study indicating that caring for children with learning disabilities might sometimes make their caregivers physically and mentally fatigued. Support from family members is therefore essential.^9^ Notably, this study found that the siblings of children with learning disabilities assisted in their education. In contrast, a previous study discovered a few problems with sibling relationships in families with children with learning disabilities.^16^ This finding might be attributed to that of previous research on individuals with learning disabilities in general. However, this study examined only families in which children with learning disabilities excelled academically. Therefore, the characteristics of the relationship between siblings might vary. If they have a good relationship, they can assist each other with their studies, consequently benefitting individuals with learning disabilities.

The strength of this study is that it is the first to collect data specifically on the parenting and education of children with learning disabilities who have good academic performance. Using a qualitative approach, the study provides insights into real-life parenting by collaborating with collections in Thailand’s provinces of various nationalities and by collecting information from the entire province. Consequently, diverse information that can be applied more extensively is generated. However, the study also has limitations related to its qualitative design. The findings may be implemented in societies with analogous circumstances to northeast Thailand (e.g. financial standing and/or family composition) but have limited applicability in regions with significantly distinct circumstances. In addition, this study collected information on a subset of parents of children with good academic performance. Since no data were obtained on children with poor academic performance, it is unknown how the parenting of this group differs from that of the group studied herein. Furthermore, the study provides information regarding the time when children with learning disabilities have good academic performance. The study did not examine children’s upbringing before the diagnosis of the learning disorder. Additionally, no details were obtained regarding children’s past academic performance or any adjustments made by their families in their parenting approach. Therefore, the impact of family support on academic performance remains uncertain.

## Conclusion

Families and caregivers of children with learning disabilities must have a thorough understanding of these disorders. Even if they are aware that their children have a learning disability, they should accept and expect that their children will have the opportunity to study to the best of their ability by enhancing their children’s learning and life skills while providing them with specialised psychological support. This approach can assist children in developing their academic skills. These findings can be used to advise families of children with learning disabilities on the significance of their role in the development of their children’s learning skills.

## Data sharing statement

The datasets are not accessible to the public. However, the data are available upon reasonable request from the corresponding author.

**How does this paper make a difference in general practice?**

- General practitioners are crucial in supporting families with children with learning impairments by providing comprehensive medical care, coordinating services, and advocating for their health and success.
- The findings can guide general practitioners make appropriate care recommendations for families with children with learning disabilities.
- Caregivers and families must set appropriate goals for learning disabled children's futures. This support is crucial for academic success.
- Older siblings and parents who live far away but can communicate via video calls, could assist primary caregivers enhance children’s academic skills.
- Prioritising the mental health of children with learning disabilities can also help them achieve academic success.
